# The role of the gut microbiome and exercise in non-alcoholic fatty liver disease

**DOI:** 10.1177/1756284820941745

**Published:** 2020-09-15

**Authors:** Veera Houttu, Ulrika Boulund, Aldo Grefhorst, Maarten R. Soeters, Sara-Joan Pinto-Sietsma, Max Nieuwdorp, Adriaan G. Holleboom

**Affiliations:** Department of Experimental Vascular Medicine, Amsterdam UMC, Location AMC at University of Amsterdam, Amsterdam, The Netherlands; Department of Vascular Medicine, Amsterdam UMC, Location AMC at University of Amsterdam, Amsterdam, The Netherlands; Department of Experimental Vascular Medicine, Amsterdam UMC, Location AMC at University of Amsterdam, Amsterdam, The Netherlands; Department of Vascular Medicine, Amsterdam UMC, Location AMC at University of Amsterdam, Amsterdam, The Netherlands; Department of Experimental Vascular Medicine, Amsterdam UMC, Location AMC at University of Amsterdam, Amsterdam, The Netherlands; Department of Endocrinology and Metabolism, Amsterdam UMC, Location AMC, Amsterdam, The Netherlands; Department of Experimental Vascular Medicine, Amsterdam UMC, Location AMC at University of Amsterdam, Amsterdam, The Netherlands; Department of Vascular Medicine, Amsterdam UMC, Location AMC at University of Amsterdam, Amsterdam, The Netherlands; Department of Experimental Vascular Medicine, Amsterdam UMC, Location AMC at University of Amsterdam, Amsterdam, The Netherlands; Department of Vascular Medicine, Amsterdam UMC, Location AMC at University of Amsterdam, Amsterdam, The Netherlands; Department of Experimental Vascular Medicine, Amsterdam UMC, Location AMC at University of Amsterdam, Amsterdam, The Netherlands; Department of Vascular Medicine, Amsterdam UMC, Location AMC at University of Amsterdam, Amsterdam, The Netherlands

**Keywords:** exercise, gut microbiome, insulin resistance, non-alcoholic fatty liver disease, obesity, type 2 diabetes mellitus

## Abstract

In recent years, the human gut microbiome has been found to influence a multitude of non-communicable diseases such as cardiovascular disease and metabolic syndrome, with its components type 2 diabetes mellitus and obesity. It is recognized to be mainly influenced by environmental factors, such as lifestyle, but also genetics may play a role. The interaction of gut microbiota and obesity has been widely studied, but in regard to non-alcoholic fatty liver disease (NAFLD) as a manifestation of obesity and insulin resistance, the causal role of the gut microbiome has not been fully established. The mechanisms by which the gut microbiome influences lipid accumulation, inflammatory responses, and occurrence of fibrosis in the liver are a topic of active research.

In addition, the influence of exercise on gut microbiome composition is also being investigated. In clinical trials, exercise reduced hepatic steatosis independently of weight reduction. Other studies indicate that exercise may modulate the gut microbiome. This puts forward the question whether exercise could mediate its beneficial effects on NAFLD *via* changes in gut microbiome. Yet, the specific mechanisms underlying this potential connection are largely unknown. Thus, associative evidence from clinical trials, as well as mechanistic studies *in vivo* are called for to elucidate the relationship between exercise and the gut microbiome in NAFLD. Here, we review the current literature on exercise and the gut microbiome in NAFLD.

## Introduction

Obesity has been on the rise since several decades and it is a major contributor to mortality across the globe.^1^ Globally, approximately 38% of adults are estimated will be overweight [Body Mass Index (BMI) above 25 kg/m^2^] by 2030. In parallel, obesity-associated disorders such as type 2 diabetes mellitus (T2DM), metabolic syndrome, and non-alcoholic fatty liver disease (NAFLD) are increasingly prevalent, as well. NAFLD can be regarded as the liver component of the metabolic syndrome and it is defined as at least 5% of fat deposit in hepatocytes. Driven by insulin resistance, it typically develops in a subset of obese patients. Currently, it is estimated that 25% of the global population has some degree of NAFLD and this number is expected to increase further with the increase of obesity and T2DM.^2,3^ The first stage of the NAFLD disease spectrum, simple steatosis, is generally believed to be rather benign in terms of liver and cardiovascular prognosis. Yet, a subset of patients develop progressive NAFLD stages, that is, inflammation [non-alcoholic steatohepatitis (NASH)] and liver fibrosis. These progressive stages are associated with an increase in mortality and morbidity, both directly in regards to liver fibrosis, cirrhosis, and hepatocellular carcinoma (HCC), as well as cardiovascular and overall mortality.^4–6^ Since NAFLD and its advanced stages are caused by a set of metabolic dysfunctions, metabolic-dysfunction-associated fatty liver disease (MAFLD) is now suggested as a new definition for the condition.^7^ In this review, the term NAFLD is still used.

There are several factors that drive obesity and NAFLD, such as a sedentary lifestyle and overnutrition. Genetic background and the gut microbiome have also been implicated in the development of NAFLD and NASH.^7,8^ Lifestyle has a major role in shaping the metabolic fluxes in key metabolically active organs such as the liver, adipose tissue and muscles. Obesity and NAFLD are, for a large part, lifestyle-induced diseases, and thus, lifestyle modifications have been recommended as a treatment.^9–11^ The two main elements of lifestyle modification are exercise and nutritional interventions and these both focus on weight management and reduction for patients with NAFLD or obesity.^9,10,12,13^ A 5–7% weight reduction is recommended to be achieved by a net calorie restriction of 500–1000 kcal/day through diet modifications alone or by combination of dietary calorie restriction and increased physical activity.

Interestingly, it has been shown that exercise alone can reduce liver fat content independent of weight loss, underscoring its beneficial effect. This liver-specific positive effect of exercise may be related to a rapid improvement in insulin sensitivity and lipid metabolism; yet, the exact mechanisms are unclear.^14^ International guidelines for obese and patients with NAFLD recommend similar amounts of physical activity as a treatment for NAFLD compared with physical activity guidelines for a healthy population.^9,10,12,13^ Current recommendations for physical activity for treatment of NAFLD and obesity according to the different international guidelines are presented in Table 1.

**Table 1.** International associations’ guidelines for exercise for the treatment of NAFLD and obesity.

Guideline	AASL 2018	EASL-EASD-EASO 2016	NICE 2016	WGO 2014
Type of exercise	Aerobic and resistance	Aerobic and resistance	No specific recommendations	Moderate exercise
Duration and frequency	150 min/week	150 min/week, 2–3 times/week	No specific recommendations	2–3 times/week

AASL, American Association for the Study of Liver Diseases; EASD, European Association for the Study of Diabetes; EASL, European Association for the Study of the Liver; EASO, European Association for the Study of Obesity; NAFLD, non-alcoholic fatty liver disease; NICE, National Institute for Health and Care Excellence; WGO, World Gastroenterology Organization.

Recent studies indicate that exercise habits not only directly influence metabolic responses, but also the gut microbiome composition and especially microbiome diversity.^15–17^ The latter two effects have recently been suggested as mechanisms by which exercise affects NAFLD, especially based on rodent studies showing modulatory effects of exercise on the gut microbiome and improvement of NAFLD.^18^ The microbiome is composed of diverse microorganisms such as bacteria, viruses, archaea, and fungi that reside in the large intestine, and contribute to several physiological processes, such as breakdown of nutrients, synthesis of vitamins, and modulation of the immune system.^19^ The gut microbiome is highly individual; but the major phyla include *Firmicutes, Bacteroidetes, Actinobacteria* and *Proteobacteria.*^20^ Throughout this review, we will focus on the bacterial component of the microbiome.

Although various studies have found that the gut microbiome can influence NAFLD, there is still debate on the mechanisms. It is suggested that either the bacteria themselves or metabolites produced by intestinal bacteria are transported over the intestinal lumen and reach the liver *via* the portal vein, where they affect hepatic metabolic and/or inflammatory pathways. Among the metabolites suggested to influence NAFLD are short-chain fatty acids (SCFAs), such as butyrate, that may protect from steatohepatitis, whereas other metabolites, such as endogenous alcohol, may promote steatohepatitis.^21,22^ However, there is conflicting evidence in regards to the specific bacterial species involved in the different disease etiologies and there is a lack of causal evidence of their effect on the diseases mentioned.^23–26^

## How the gut microbiome can shape obesity and NAFLD

There have been multiple efforts to identify a signature change in the gut microbiome of patients with NAFLD compared with healthy controls. Although the results of these studies cannot be compared side by side, it is evident that the microbiome associated with NAFLD has lower richness, but the relative difference in bacterial composition between NAFLD patients and healthy controls is not yet clear. For instance, while some studies found that the gut microbiome of NAFLD patients has an increased abundance of *Fusobacteria* and reduced amounts of *Oscillospira* and *Ruminococcus*,^27^ children with NAFLD have been shown to have more *Ruminococcus* and *Oscillospira* than healthy children.^28^ It is clear that further research is needed to determine what differentiates the gut microbiome in patients with NAFLD from that of healthy subjects. Therefore, it is also not known what drives the differences in the gut microbiome compositions between patients with NAFLD and healthy controls, and how this affects disease state or progression. It has been proposed that the gut microbiome can increase energy uptake and initiate inflammation, two factors contributing a dysfunctional metabolism,^20,26,29,30^ but the microbiome might also affect other processes associated with NAFLD such as insulin resistance, and metabolism of bile acids and choline.^31^

Some of the first indications of the role of the gut microbiome in accumulation of fat in the liver was found in 1982 when researchers found that treatment with the antibiotic metronidazole after a gastric bypass operation resulted in a reduction in hepatic steatosis.^32^ Similar results were also seen in rats, where metronidazole and tetracycline diminished liver damage after surgery.^33^ Mouzaki *et al*.^30^ characterized the gut microbiome composition of healthy controls, NAFLD subjects, and NASH subjects, and found that those with NASH had a lower abundance of *Bacteroidetes* compared with subjects with simple steatosis or healthy subjects, while the intestinal microbiome did not differ between NAFLD subjects and controls. However, Raman *et al*.^34^ showed that there were significant differences in the fecal microbiome between NAFLD subjects and healthy controls, with higher abundance of *Lactobacillus* species and certain members of the *Firmicutes* phylum in NAFLD subjects. Schwimmer *et al*.^35^ also saw a lower microbial diversity (alpha diversity) in children with NAFLD compared with controls. Of interest, *Helicobacter pylori* may in some cases contribute to NAFLD.^36^ *H. pylori* has been seen in severely obese subjects,^37^ and it is associated with insulin resistance, which can be a driver of NAFLD. Recently, *H. pylori* has also been independently associated with advanced stages of NASH.^38^ Some suggested mechanisms are that it may inhibit the release of leptin from white adipose tissue, promote liver stearoyl-CoA desaturase-1 (SCD1) and increase hepatic fatty deposition. The effect *of H. pylori* on NAFLD could also be driven by the effects this bacterium has on gut dysbiosis, endotoxemia and dyslipidemia.^36^

The reviewed human studies that report differences in the general gut microbiome composition between patients with NAFLD and control subjects are summarized in Table 2. From these studies it is clear that the richness and diversity of the gut microbiome is lower in patients with NAFLD than in healthy controls. More details are provided in Table 3 which shows a summary of studies that report changes on the phyla level of the gut microbiome in NAFLD, and in Table 4 that recapitulates the genera level changes. Finally, Table 5 holds the studies with species-specific reports on changes in NAFLD populations.

**Table 2.** Summary of reviewed studies reporting changes in the gut microbiome composition in patients with NAFLD compared with healthy control subjects.

Microbial entity	Effect	Population	Reference
Alpha diversity	Decreased	NAFLD	Del Chierico *et al*.^28^
	Decreased	Pediatric NAFLD	Schwimmer *et al*.^35^
Richness	Decreased	NAFLD	Kim *et al*.^27^
*Bacteroidetes*/*Firmicutes* ratio	Decreased	NAFLD	Da Silva *et al*.^39^

NAFLD, non-alcoholic fatty liver disease.

**Table 3.** Summary of reviewed studies reporting phylum level differences in patients with NAFLD.

Phyla	Effect	Population	Reference
*Firmicutes*	Increased	NAFLD	Raman *et al*.^34^
	Decreased	NASH	Zhu *et al*.^40^
*Actinobacteria*	Increased	Pediatric NAFLD	Del Chierico *et al.^28^*[Bibr bibr24-1756284820941745]
	Decreased	NASH	Zhu *et al*.^40^
*Bacteroidetes*	Decreased	NASH compared with SS and controls (no difference between SS and controls) Independent of BMI and dietary fat intake	Mouzaki *et al*.^30^
	Decreased	Pediatric NAFLD	Del Chierico *et al*.^28^
	Increased	NASH	Zhu *et al*.^40^
*Proteobacteria*	Increased	NASH	Zhu *et al*.^40^

BMI, body max index; NAFLD, non-alcoholic fatty liver disease; NASH, non-alcoholic steatohepatitis; SS, simple steatosis.

**Table 4.** Summary of reviewed studies reporting genus level differences in patients with NAFLD.

Genus	Effect	Population	Reference
*Oscillospira*	Decreased	NAFLD	Kim *et al*.^27^
	Decreased	Pediatric NAFLD	Del Chierico *et al*.^28^
*Coprococcus*	Decreased	NAFLD	Kim *et al*.^27^
	Decreased	NASH and SS, independent of BMI and IR	Da Silva *et al*.^39^
*Ruminococcus*	Decreased	NAFLD	Kim *et al*.^27^
	Decreased	NASH and SS, independent of BMI and IR	Da Silva *et al*.^39^
	Increased	Pediatric NAFLD	Del Chierico *et al*.^28^
*Lactobacillus*	Increased	NAFLD	Da Silva *et al*.^39^
	Increased	NAFLD	Raman *et al*.^34^
*Bradyrhizobium*	Increased	Pediatric NAFLD	Del Chierico *et al*.^28^
*Anaerococcus*	Increased	Pediatric NAFLD	Del Chierico *et al*.^28^
*Peptoniphilus*	Increased	Pediatric NAFLD	Del Chierico *et al*.^28^
*Dorea*	Increased	Pediatric NAFLD	Del Chierico *et al*.^28^
	Increased	NAFLD	Raman *et al*.^34^
*Robinsoniella*	Increased	NAFLD	Raman *et al*.^34^
*Roseburia*	Increased	NAFLD	Raman *et al*.^34^
*Oscillibacter*	Decreased	NAFLD	Raman *et al*.^34^
*Escherichia*	Increased	NASH	Zhu *et al*.^40^

BMI, body max index; IR, insulin resistance; NAFLD, non-alcoholic fatty liver disease; NASH, non-alcoholic steatohepatitis; SS, simple steatosis.

**Table 5.** Summary of reviewed studies reporting species level microbiome differences in patients with NAFLD.

Species	Effect	Population	Reference
*Propionibacterium acnes*	Increased	Pediatric NAFLD	Del Chierico *et al*.^28^
*Klebsiella pneumoniae*	Increased	NAFLD	Yuan *et al*.^21^
*Faecalibacterium prausnitzii*	Decreased	NASH and SS, independent of BMI and IR	Da Silva *et al*.^39^
*Helicobacter pylori*	Increased	Obese subjects (some with NASH)	Emile *et al*.^37^ and Doulberis *et al*.^38^

BMI, body max index; IR, insulin resistance; NAFLD, non-alcoholic fatty liver disease; NASH, non-alcoholic steatohepatitis; SS, simple steatosis.

It is clear that the gut microbiome is strongly associated with NAFLD, and several studies demonstrate multiple changes in the microbiome of NAFLD patients compared with healthy subjects. However, there are some conflicting results, and this may largely be due to the high individuality of one’s gut microbiome. To summarize the only consistent effect on the phyla level is an increase in *Proteobacteria* in NAFLD subjects. Interestingly, members of this phylum carry lipopolysaccharide (LPS) in their outer membranes, which may be a player in NAFLD. This phylum has also previously been associated with inflammation.^41^ *Bacteroidetes* has mainly been reported as decreased in NAFLD, and this is a highly abundant mostly symbiotic member of the healthy gut microbiome.^42^

One reason for the inconsistent findings on the phyla level may be due to the fact that one phylum typically comprises many different species, and one phylum may be dominated by certain species in one subject but dominated by other species in another subject. Thus, the phyla level can be subject to a noisy signal of the changes at other taxonomical levels. The evidence on genus and species level is more consistent, pointing to an increase in the relative abundance of the genera *Lactobacillus, Bradyrhizobium, Anaerococcus, Peptoniphilus, Dorea, Robinsoniella, Roseburia* and *Escherichia* in subjects with NAFLD. The genera *Oscillospira, Oscillibacter, Coprococcus*, and *Ruminococcus* have a rather low abundance in NAFLD subjects, albeit that the latter was reported to be increased in a pediatric population with NAFLD. There are not a lot of studies reporting species level differences, though *Propionibacterium acnes, Klebsiella pneumoniae* and *H. pylori* were all reported as increased in subjects with NAFLD or obesity, and *Faecalibacterium prausnitzii* was reported as decreased. It is important to note that many of these studies have not investigated the functional capability of the microbiome. The same species may exhibit quite different functional genes in different environments. This could be another reason why there are conflicting results in the current microbiome analyses in NAFLD. Thus, in general analyses that have shotgun metagenome, sequencing is preferable over 16s amplicon sequencing; of the studies mentioned in Tables 2–5, only Raman *et al*. and da Silva *et al*. had performed metagenome sequencing.^34,39^

In the last few years, several studies focusing on microbiome and metabolome data have made it evident that many factors are involved in the association of hepatic lipid content and the gut microbiome. These factors include the direct effects of the microbes themselves, such as by facilitating energy harvest and their metabolic mechanisms, as well as their secreted metabolites that, *via* the portal vein, affect hepatic metabolic pathways. Among these factors are the SCFAs, LPS, and ethanol. In the remainder of this review, we will discuss several roles of the intestinal microbiome in influencing hepatic lipid metabolism.

### The gut microbiome can increase the energy harvest of the host

A direct link between the gut microbiome and the hepatic lipid content is provided by the fact that certain microbiota can facilitate energy harvest. In various studies that have mainly been performed in mice, the effect of the microbiome on obesity was shown to be mediated *via* an enhanced energy harvest, and some of these studies have also shown an effect on NAFLD/hepatic lipid accumulation.

Among these studies, those with germ-free mice have been especially instrumental to show a role of gut microbiota in obesity and NAFLD. For instance, introducing the microbiota from conventionally raised animals into germ-free mice resulted in a 60% increased body fat content, despite reduced food intake. The body fat increase appeared to be due to an elevated absorption of monosaccharides in the gut lumen. In line, other studies also report that obese mice extracted more energy from the diet, and this effect could be transferred to germ-free mice upon fecal transplantation of the fecal content from obese mice.^25,43^

Alongside, diet composition also plays an essential role in weight gain of germ-free mice. Several studies find that germ-free mice do not develop obesity on a Western-type diet (WTD); however, on the high-fat diet they do increase more in body weight and fat, compared with conventional mice.^44–46^ On the low-fat diet; however, there was no difference between germ-free and conventional mice with respect to body weight. Thus, the fact that the mice are germ-free is not, in itself, protective of obesity, but rather in combination with the composition of the diet, it may influence weight gain.

The specific mechanisms underlying the interaction between the gut microbiome and the diet are still being investigated. Germ-free mice on a WTD had increased levels of fasting-induced adipose factor (Fiaf), as well as a higher adenosine monophosphate (AMP)-activated protein kinase (AMPK) activity as compared with mice with a gut microbiota on the same diet.^44^ These factors are not related; however, they both contribute to an elevated hepatic oxidation of fatty acids. Despite the fact that germ-free mice on high-fat and WTDs did have an increased messenger ribonucleic acid (mRNA) expression of Fiaf compared with conventional mice on the same diet, no significant changes in circulating Fiaf were seen. Thus, it seems as if Fiaf is not the causative agent for an increased body fat content through the gut microbiome.^46^ The abovementioned evidence all relates to obesity, but it may be different for liver fat. One study published in 2013 did find that germ-free mice that received fecal transplantation from mice with hyperglycemia developed hepatic steatosis, at least partly due to an increased *de novo* lipogenesis (DNL).^47^

It is always a challenge to translate murine evidence to humans for a number of reasons (e.g. mice are coprophagic, and the microbiomes between humans and mice are vastly different).^48,49^ It is also important to remember that the majority of the studies mentioned previously investigate general fat storage, not specifically hepatic fat. Clearly, human validation of these animal models is called for. However, some human evidence exists. One study investigated the result of increased caloric intake on the composition of the gut microbiome in 12 lean and 9 obese individuals. An increased caloric intake in lean individuals resulted in a decreased fractional energy loss from the stool. However, there was no difference in the obese group. There was no difference in stool energy excretion between lean and obese subjects on the low-calorie diet.^50^ Our recent fecal microbiota transplantation (FMT) study in which we compared feces from post-bariatric surgery donor with feces from metabolic syndrome patients revealed that they had no significant effect on metabolic parameters.^51^

Other physiologically important factors in weight management and energy metabolism are adipokines, endocrine factors secreted by adipose tissue, of which leptin is a well known. Adipokines play a central role in energy metabolism, but perhaps also in the interaction between the gut microbiome and energy metabolism. Leptin-deficient mice develop an obese phenotype which can be transferred with a fecal transplant into germ-free mice.^25^ Also, leptin influences liver pathophysiology by increasing liver fibrinogenesis in mice, but these findings have not been reproduced in humans.^52^

Taken together, there is mounting evidence from mouse studies indicating the importance of diet composition and the gut microbiome on weight gain, and this likely also holds true for humans as well. Yet, the mechanistic insights here are sparse and might be pointing towards an effect on (hepatic) fatty-acid oxidation. Also, increased energy harvest by the gut microbiome is likely a major driving force in NAFLD.

#### Short-chain fatty acids

SCFAs such as butyric, acetic, and propionic acid are produced by bacterial fermentation of nutritional fibers and other macronutrients in the gut.^20^ SCFAs function as energy substrates but they can also modulate energy metabolism, for instance, by suppression of adipocyte leptin secretion, resulting in induced satiety and hence reduced food intake.^53^ Thus, SCFAs can link microbiome and host metabolism in intricate ways.

The evidence of SCFAs influence on NAFLD in mice is quite convincing. One study found that mice receiving supplementation of acetic acid had decreased hepatic lipid accumulation.^54^ Several other studies in which mice were fed SCFAs also found a decrease in hepatic fat, alongside activation of intestinal gluconeogenesis, or reduced energy intake and protection against obesity.^55–57^ The possible mechanisms are numerous and likely depend on the subtype of SCFAs and on factors such as diet and microbiome composition. It has been indicated that acetic acid supplementation to rats resulted in a decreased hepatic mRNA expression of crucial lipogenic genes such as fatty-acid synthase (*FAS*), acetyl coenzyme A carboxylase (*ACACA*) and malic enzyme (*ME*), suggesting reduced hepatic DNL.^58,59^ It appears that peroxisome proliferator-activated receptor-γ (PPAR-γ) may be involved in hepatic lipid accumulation because SCFAs had no effect on hepatic steatosis when fed to mice deficient for hepatic PPAR-γ.^55^ Other mechanisms by which SCFAs may be involved in reduced hepatic lipid accumulation are by suppressing appetite or by increasing fat oxidation *via* the activation of brown adipose tissue [measured by the expression of uncoupling protein 1 (UCP-1)]. These both may lead to less availability of fatty acids and therefore decreased fat storage in the liver.^55–57,60^

Currently, no studies have investigated the effects of SCFAs on NAFLD/NASH in humans; hence, only circumstantial or indirect evidence exists.^54^ However, a well-designed randomized crossover control trial with approximately 60 subjects showed that propionate supplementation prevented weight gain in obese humans.^61^ In relation to this, several studies indicate that supplementing with fibers in the diet can increase satiety in humans; however, they do not necessarily investigate the conversion into SCFAs.^62,63^ A 6-week trial with fiber supplementation found a suppressing effect on appetite, though no effect on body weight or adiposity.^64^ Although markers of NAFLD were not determined, a study found that oral butyrate supplementation improved glucose metabolism in lean subjects but not in obese subjects. The human data suggest the fecal SCFAs have differential effects in healthy *versus* diseased (obese) subjects.^65^

Thus, SCFAs may hold promise in their effect on human energy metabolism, though the effect may be limited in the obese population and their effect in patients with NAFLD is still to be studied. Interestingly, certain SCFAs can inhibit histone deacetylases (HDACs), and several HDACs are believed to play a role in the pathogenesis of NAFLD.^66,67^ Altogether, there lays a great potential in further elucidating the mechanisms of SCFAs in energy metabolism in NAFLD subjects.

### Gut permeability

#### Portal vein connection

The liver is connected to the intestine through the portal vein, and approximately 70–75% of the blood that reaches the liver is drawn from veins surrounding the gut and transported through the portal vein.^68^ This means that bacterial components and metabolites can readily be directed from the gut to the liver. It has been estimated that more than 300 volatile organic compounds are produced by the gut that reach the liver where they might affect metabolic processes.^69^ Thus, the permeability of the gut might be crucial in controlling the amount of bacterial compounds that can directly reach the liver. Currently, there is no causal evidence of a ‘leaky gut,’ but we will cover some of the small murine and human studies that have been reported.

A hypothesis that is based on this liver connection suggests that due to increased gut permeability, microbe-derived substances can reach the liver, to a higher degree. These substances include the abovementioned SCFAs, but also metabolic compounds such as choline, ethanol, bile acids, and small bacterial components such as LPS or flagellins. An increased gut permeability was seen in mice fed a high-fat diet.^70^ Even more interestingly, the degree of liver steatosis correlated with gut permeability in a study of patients with biopsy-proven NAFLD. However, the presence of NASH was not linked to gut permeability. This is an interesting finding, and the evidence seems quite robust considering the study size (35 subjects with NAFLD, compared with 27 with celiac disease, and 24 healthy subjects).^71^ In contrast, a more recent review showed that less than half the patients with NAFLD had increased gut permeability.^72^ Thus, even though the translocation of molecules due to a ‘leaky gut’ may exacerbate the disease in a subset of subjects, it is not the only driving force in NAFLD. It is still of major interest to elucidate which patients with NAFLD have leaky gut and which do not, as this may seem to be an important initiating factor in the pathogenesis of NAFLD for many patients. Finally, an increased gut permeability seems to be one of the first mechanisms that allow for further injury by microbial produced compounds, thus it may be a key factor in the connection of the gut microbiome with NAFLD. Unfortunately, there is no consensus on how to assess gut permeability, and causal evidence is severely lacking. Potentially, a combination of several assays is the best way forward.^73^

#### Bile acids

Bile acids have diverse functions that originally were thought only to be the emulsification of lipids and lipid-soluble vitamins in the gut to facilitate intestinal uptake, as well as the secretion of endogenous metabolites in the hepatobiliary system. Nowadays it is acknowledged that bile acids also have an important role in regulating lipid, carbohydrate, and cholesterol metabolism, and signaling pathways.^74^ Bile acids can be categorized into primary and secondary bile acids. Primary bile acids are conjugated products from the liver bile acid pool, while secondary bile acids have been deconjugated by the gut microbiome. A change in their metabolism and composition can lead to damaging effects on the liver.^27,74^ It is also thought that farnesoid X receptor (FXR), a nuclear transcription factor of which bile acids are endogenous ligands, might play a role in NAFLD due to its impact on the oxidation of lipids and affect the metabolism of cholesterol and carbohydrates.^74^ Furthermore, deletion of FXR leads to an increased inflammation in the liver in a WTD mice model^75^ and development of HCC markers induced by a bile acid (cholic acid),^76^ which indicates its pivotal role on the bile acid metabolism and the liver.

The gut microbiome can *via* diverse enzymatic mechanisms regulate the size as well as the composition of the bile acid pool, which can have several effects on the signaling properties of bile acids.^27^ Vice versa, bile acids can also shape the microbiome composition, for example, by production of antimicrobial compounds, or by their role as nutrients for certain bacteria. FXR knockout (FXR KO) mice fed a WTD showed different composition in gut microbiota, namely reduced *Firmicutes* and increased *Proteobacteria* compared with controls on either WTD or control diet.^75^ Also, the WTD fed FXR KO mice had an increased serum LPS compared with FXR KO on control diet, and compared with controls on either diet. The increased serum LPS was rescued by antibiotic treatment, indicating the link between gut bacteria, derived metabolites, and hepatobiliary system. In parallel the wild type (WT) and FXR knockout mice developed NASH when fed the WTD (the FXR KO mice had more inflammatory phenotypes than WT mice), which was halted with antibiotic treatment suggesting a link between inflammation in the liver, bile acids, and gut microbiome.

There is some evidence of the relation between bile acids, the microbiome and NAFLD in humans. Several microbial pathways related to bile acid biosynthesis were detected in high abundance in subjects with NAFLD compared with controls. Furthermore, subjects with NAFLD and particularly NASH have been shown to have an elevated secondary bile acid production. However, it appears that subjects with cirrhosis have less fecal bile acids, which can be seen as a proxy for the decrease of the conversion of primary to secondary bile acids. The effects on the bile acid pool may thus be another pathway for further injury to the liver, and according to some studies may contribute to the persistence of NAFLD.^27^

#### Ethanol produced by the gut microbiome

Ethanol is an important contributor of alcoholic fatty liver disease (AFLD) which can ultimately lead to similar endpoints as NAFLD, namely fibrosis, cirrhosis, and liver failure.^77^ It has been demonstrated that the gut microbiota can produce both ethanol and methanol, and since patients with NAFLD have similar histopathology compared with AFLD subjects, a hypothesis evolved that the endogenous alcohol production by the gut microbiota may be a major factor in the pathogenesis of NAFLD.^78–80^ This hypothesis is supported by a study that found genetically obese mice had an increased intestinal ethanol production compared with lean mice.^81^

Several studies reported higher serum ethanol levels in subjects with NAFLD compared with controls, and one of these studies also found an increased intestinal permeability in these subjects. Interestingly, one study compared healthy controls, obese subjects without NASH, and subjects with NASH, and found higher blood ethanol levels in subjects with NASH compared with the other two groups; thus, high plasma ethanol concentrations appear to be specific to NAFLD, not obesity.^40,82^ In addition to this, it appears that the endogenous ethanol in the blood is increased in subjects with NASH compared with simple NAFLD/steatosis.^83^ One study found increased endogenous ethanol in children with NAFLD and suggested an association to specific bacterial taxa in the gut.^84^ In contrast, a study published in 2013 did not find a significant difference in ethanol when comparing the volatile fecal compounds in patients with NAFLD and healthy controls. However, they did not measure the concentration of alcohol in breath or blood.^34^ A very recent study found that a strain of high-alcohol-producing *K. pneumoniae* was associated with a majority of subjects with NAFLD in a Chinese cohort. Interestingly, NAFLD was induced in mice when they received fecal microbiota that contained this particular strain.^21^

Despite these convincing studies with the *K. pneumoniae* strain, it has been suggested that increased ethanol levels in patients with NAFLD may be due to insulin-dependent impairments of the alcohol dehydrogenase pathway rather than an increase in endogenous alcohol production from the gut.^85^ Thus, endogenous ethanol production by gut microbiota seems very likely to be one of the major culprits in the development of liver steatosis in some, but surely not all NAFLD patients.

#### Endotoxemia

The term ‘metabolic endotoxemia’ is used to describe an increase in LPS-containing bacteria in the gut and an increased LPS concentration in plasma, this was found in mice fed a high-fat diet.^62^ There are multiple studies suggesting that endotoxemia is a part of the pathophysiology of obesity; however, studies specific to NAFLD are lacking. In addition to this, the assays for LPS in plasma can be unreliable.^86^ There is a need for more rigorous analyses of the assays used in these studies to improve the hypotheses and research on endotoxemia.

#### Choline

Choline is a compound that for decades has been implicated with fatty liver. Rats fed a choline-deficient diet develop steatosis due to dysfunctional hepatic lipoprotein metabolism.^87^ Since phosphatidylcholine is a major component of lipoproteins, choline deficiency leads to improper formation of lipoproteins. Choline deficiency also leads to a hepatic uptake of free fatty acids and therefore triglyceride build-up in the liver, but this can be reversed shortly after returning choline to the diet.^88^ Providing mice with antibiotics upon a phosphatidylcholine challenge affects choline metabolism by suppression of conversion of phosphatidylcholine into trimethylamine-*N*-oxide (TMAO) by the gut microbiota, suggesting that some effects of choline on NAFLD may be regulated *via* the gut microbiome.^89^ Indeed, a small study of 15 subjects placed on a choline-deficient diet detected specific microbiota markers of NAFLD in their feces.^90^ Other supporting evidence comes from patients with parenteral nutrition who suffer from choline deficiency and therefore had significantly increased liver enzymes and liver steatosis.^91,92^ To conclude, the evidence of the effects of choline deficiency on NAFLD are quite robust, and it seems likely that they are regulated by the gut microbiome; however, the specific pathways and microbes responsible for this effect remain to be identified.

## How exercise affects NAFLD

Recent rodent studies investigating the effects of exercise on NAFLD indicate that exercise improves liver status through mechanisms that include hepatic or peripheral lipid metabolism, insulin sensitivity, and inflammatory responses in the liver. These effects of exercise on the liver can be mediated *via* connections with other organs, including adipose and muscle tissues. As with many of the previous aspects discussed in this review, verification of the role of exercise on hepatic fat content in humans is warranted.^93^ Next, we review the evidence on these mechanisms in order to facilitate the understanding of the connection between the gut microbiome, exercise, and NAFLD further on in this review.

### Hepatic and peripheral lipid metabolism

Various randomized controlled trials (RCTs) have reported a beneficial effect of exercise on hepatic steatosis.^94–99^ A recent meta-analysis of 17 such RCTs in NAFLD patients revealed a significant decrease in intrahepatic triglyceride (IHTG) content independently of weight reduction, upon exercise. On average, a 3.31% decrease in IHTG content was measured by magnetic resonance spectroscopy (MRS) or by liver biopsy.^14^ Since there is a big variation in the type, duration, and frequency of exercise, it is still unclear what kind of exercise is the most beneficial. However, the latest literature suggests that low-intensity training, and especially resistance training (RT), is not sufficiently effective to reduce IHTG content.^100^ It is obvious that there is a need for more studies to further conclude which is the most beneficial type and frequency of exercise in the management of NAFLD. Additionally, the absolute decrease in IHTG content that is seen in most studies is clinically rather irrelevant due to small effect size, but it can open future insights to the pathophysiology of NAFLD and its progressive stages.

As mentioned, it is recognized that the impact of exercise on steatosis is driven by mechanisms bypassing weight reduction, since most clinical studies report improvement on IHTG content even in the absence of significant weight loss.^97,101–103^ However, even if there is no weight change upon exercise, the body composition is altered, for example, reduced fat mass, in the majority of exercise studies, the reduction in fat mass may be important when interpreting the impact of exercise on liver metabolism, since loss of adipose tissue induces improvements in metabolic health which cannot be overlooked.^104^ In general, visceral and subcutaneous adipose tissue volumes are decreased upon exercise, albeit that these effects were most pronounced in studies with significant weight reduction, not in studies without weight reduction.^98,105^ Thus, it seems as if changes in body weight and body composition together may partly drive the beneficial effects of exercise on NAFLD.

Exercise regulated lipid metabolism in parallel with an improvement in NAFLD, as was shown in a study in which rats with NAFLD received 6 weeks of training.^18^ After these 6 weeks of training, the improved liver histology was accompanied by reductions in the hepatic mRNA expression of gene-encoding enzymes involved in lipogenesis, such as sterol regulatory element-binding protein 1c (*Srebp1c*), CCAAT/enhancer-binding protein alpha (*Cebpa*) and the fatty-acid transporter (*Cd36*). In another study, 20 male C57BL/6J ApoE-KO mice that were fed a high-fat diet underwent a swimming exercise for 12 weeks. This exercise resulted in improvements in insulin sensitivity, and in reductions in lipid concentrations in plasma, as well as in liver.^106^ Exercise increased the relative protein expression of enzymes involved in hepatic fatty-acid oxidation, such as PPAR-γ, medium-chain acyl-CoA dehydrogenase (MCAD) and carnitine palmitoyltransferase 1 (CPT-1). In the ApoE-KO mouse model, the exercise results in more fatty-acid oxidation. This suggests that exercise enhances hepatic fatty-acid oxidation resulting in a reduced liver lipid content and increased insulin sensitivity.

### Hepatic and/or peripheral insulin sensitivity

Exercise likely improves hepatic as well as peripheral insulin sensitivity of obese insulin-resistant subjects. Improved insulin sensitivity will likely cause an increased glucose uptake in muscles and liver for glycogen synthesis, but also the inhibition of glucose secretion and stimulation of DNL by insulin is likely to be increased through the improvement of insulin sensitivity.^98,107^ As seen with the exercised ApoE-KO mice mentioned above, exercise might stimulate the hepatic oxidation of fatty acids, affecting not only the hepatic fat content, but also, as a consequence, hepatic insulin sensitivity. Importantly, exercise might result in a decreased lipolysis, as a reflection of improved insulin sensitivity in adipose and other tissues; hence, reduced flux of fatty acids from tissues to the liver and thereby reduced accumulation of triglycerides. Moreover, after a high-intensity exercise training for 4 weeks, postprandial circulating lipid peroxidation levels were reduced,^101^ suggesting hepatic oxidative stress was reduced by aerobic exercise.^108^ This, altogether, would indicate improvements of liver status. However, the evidence of impact of different types of exercise on insulin sensitivity is controversial; sprint–interval training did not have significant impact on either peripheral or hepatic insulin sensitivity, but other types of aerobic exercise improved peripheral insulin sensitivity.^98,107^

### Inflammatory responses

Inflammation is the hallmark of the advanced stages of NAFLD which eventually led to fibrosis in the liver. Oh *et al*.^103^ studied the effect of high-intensity interval aerobic training (HIAT), RT or moderate-intensity continuous training (MICT) on liver fat, as well as liver stiffness as assessed by vibration-controlled transient elastography (VCTE), a validated non-invasive parameter of NAFLD-related fibrosis.^109^ Hepatic fat was reduced in both HIAT and MICT groups, but reduction of hepatic fat stiffness was only found in the HIAT group. These differences could be due to differences in intensity of exercise. Another study did not find any significant improvements in liver fibrosis assessed by VCTE when comparing baseline and endpoint upon exercise.^110^ On the other hand, promising results were presented from a 12-week aerobic-exercise intervention with significantly reduced fibrosis at endpoint biopsies, as well as improved liver stiffness scores measured with VCTE, and improved steatosis measured with controlled attenuation parameter (CAP).^111^

With respect to the effect of exercise on inflammation and hepatic stiffness, it has been suggested that exercise restores the phagocytic function of damaged Kupffer cells, hepatic macrophages that play a central role in the pathophysiology of hepatic inflammation,^112^ which may in turn decrease inflammation and fibrosis.^103^ However, the effect of exercise on more progressive forms of NAFLD such as steatohepatitis and fibrosis, has been shown to be quite modest; though, exercise may be less efficient in this group of patients because they may exhibit a decreased maximal workload capacity compared with patients with liver steatosis due to increased diastolic dysfunction.^113^ In a mouse model after an intense 8-week exercise intervention, high-fat induced NASH mice showed histological improvements in liver injury and also in the structure and enzyme activities of hepatic mitochondria.^114^ Altogether, more insights into the effect of exercise specifically on non-alcoholic-related liver outcomes are called for.

## How exercise shapes the gut microbiome

Exercise has not only been reported to affect body weight and hepatic fat content in various animal models, as well as in some human studies, it also has profound effects on the gut microbiome. The effects can be detected either on the compositional level or the metabolites produced by the gut microbiome. However, the exact role of the gut microbiome in controlling or mediating the effects of exercise in (obese) subjects is still under investigation. Table 6 summarizes the studies on the effect of exercise on the gut microbiome in obese populations. We will highlight some of these studies.

**Table 6.** Summary of reviewed studies reporting effects of exercise on the human gut microbiome in patients with overweight, obese and diabetes.

Microbial entity		Effect	Population, exercise duration and type	Reference
**Alpha diversity**		No change	Obese and lean subjects 6 weeks’ endurance exercise	Allen *et al*.^115^
		No change	Obese subjects 6 weeks’ endurance exercise	Munukka *et al*.^15^
		Increased	Obese and overweight subjects 3 months’ aerobic exercise with different intensities	Kern *et al*.^17^
***Bacteroidetes/Firmicutes* ratio**		No change	Obese subjects 6 weeks’ endurance exercise	Munukka *et al*.^15^
		Decreased	Prediabetics and T2DM 2 weeks’ MICT or SIT	Motiani *et al*.^16^
**Phyla**	Overall	No change	Obese subjects 6 weeks’ endurance exercise	Munukka *et al*.^15^
	*Bacteroidetes*	Increased	Prediabetics and T2DM 2 weeks’ MICT or SIT	Motiani *et al*.^16^
	*Verrumicrobia*	Increased	Obese subjects 6 weeks’ endurance exercise Independent of age, weight, fat%, energy and fiber intake	Munukka *et al*.^15^
	*Proteobacteria*	Decreased	Obese subjects 6 weeks’ endurance exercise Independent of age, weight, fat%, energy and fiber intake	Munukka *et al*.^15^
**Genus**	*Akkermansia*	Increased after exercise	Obese subjects 6 weeks’ endurance exercise Independent of age, weight, fat%, energy and fiber intake	Munukka *et al*.^15^
	*Blautia, Clostridium*	Decreased after exercise	Prediabetics and T2DM 2 weeks’ MICT or SIT	Motiani *et al*.^16^
**Species**	*Clostridiales* spp., *Lachnospira* spp., *Roseburia* spp., family *Lachnospiraceae* unclassified, *Faecalibacterium* spp.	Increased	Obese and lean subjects Independent of BMI 6 weeks’ endurance exercise	Allen *et al*.^115^
	*Anaerotruncus colihominis, Bilophila*, wadsworthia, *Alistipes putredinis, Streptococcus mitis* groups	Deceased	Prediabetics 12 weeks’ aerobic and resistance exercise HIIT	Liu *et al*.^116^
	*Phascolarctobacterium succinatutens, Bacteroides xylanisolvens*	Increased	Prediabetics 12 weeks’ aerobic and resistance exercise HIIT	Liu *et al*.^116^

fat%, fat percentage; HIIT, high-intensity interval training; MICT, moderate-intensity continuous training; SIT, sprint–interval training; T2DM, type 2 diabetes mellitus.

The gut microbiota alpha diversity was increased in obese and overweight subjects after a 3-month-period of vigorous-intensity exercise compared with controls with habitual daily living.^17^ Changes were also detected in beta diversity in groups with vigorous-intensity, moderate-intensity exercise and activity done by commuting by bike compared with the habitual-daily-living control. However, there were no changes in bacterial genera. Another study found a decrease in the *Firmicutes/Bacteroidetes* ratio in patients with prediabetes and T2DM after short-periods of sprint–interval training.^16^ Also, a significant decrease in the abundance of the genera *Blautia* and *Clostridium* was found. The authors of this latter article suggest that exercise has a reducing effect on endotoxemia measured by intestinal lipopolysaccharide-binding protein, but the study did not have a proper sedentary control group without any exercise; the participants were either divided into sprint–interval or MICT groups. In contrast, however, another study did not find any changes in alpha diversity or in the phylum level after a 6-week endurance training program, but increased relative abundances of *Verrucomicrobia* and decreased *Proteobacteria* were found, suggesting that these two phyla may respond to exercise, at least in obese women.^15^

Not only the gut microbiome, but also metabolites produced by the gut microbiome have been reported to be affected by exercise. For instance, a 6-week endurance training in 14 obese and 18 lean subjects exhibited changes in beta diversity in the obese subjects; but fecal SCFAs, including butyrate, were increased only in lean subjects after the exercise.^115^ Yet, the abundance of butyrate-producing genera were increased, irrespective of BMI. A recent study in patients with prediabetes reported significant differences in the gut microbiome composition after a 12-week aerobic-exercise intervention, but only when subjects were divided into responders and non-responders based on their response to the exercise program with respect to changes in insulin sensitivity. Exercise-mediated effects on the gut microbiota composition correlated strongly with insulin sensitivity and blood-sugar regulation. Compositional differences in the gut microbiota were also seen without the groupings. Nevertheless, fecal metabolites differentiate significantly between these two groups; for instance, responders had significantly decreased abundance of SCFAs and branched-chain amino acids after the exercise protocol, whereas non-responders had the opposite effect upon exercise. These results further support that exercise might mediate effects on human metabolism through changes in the gut microbiome.

Studies on exercise in rodent NAFLD models investigating the gut microbiome and its metabolites are not so numerous. Among the few is one that shows that high-intensity exercise training for 6 weeks improved gut microbiome diversity but not body weight of C57BL/6 mice fed a high-fat diet.^117^ More specifically, the training increased alpha diversity and the *Bacteroidetes*/*Firmicutes* ratio. In contrast, another study with the same mice model showed a significant decrease in the ratio of *Bacteroidetes* and *Firmicutes* midway of a voluntary 12-week exercise intervention, while after the whole voluntary exercise period, the ratio was slightly increased, albeit its statistical significance. The body weight was also affected by exercise.^118^ Interestingly, low or moderate training had only minimal impact in modulating the gut microbiome in mice fed high-fat diet after 8 weeks of treadmill training.^119^ The abundance of *Proteus* and *Vagococcus* were changed upon exercise. However, the body composition was changed by the exercise. In diabetic rats, high-intensity interval training (HIIT) improved metabolic health without reshaping the gut microbiota.^120^

## The interaction of the gut microbiome and exercise on NAFLD

To the best of our knowledge, the impact of exercise specifically on the gut microbiome and its subsequent effects on NAFLD has not been studied in humans. Thus, when researchers suggest that exercise might affect NAFLD (in part) due to modulation of the intestinal microbiome, this is attributed to effects seen on obesity, an important contributor to NAFLD. Interestingly, there is only one rodent study addressing the modulatory effect of exercise on NAFLD through gut microbiome.^18^ In this study, the effect of a controlled exercise program was comprehensively investigated on the gut–liver axis in juvenile rats fed a high-fat diet with promising results, when the mice were introduced on an exercise program with aerobic and RT for 5 weeks after the development of features for obesity and NAFLD, which were induced by a 6-week high-fat diet before the start of the exercise program.

A recent study in which NAFLD was induced in rats by feeding them a high-fat diet demonstrated that exercise had effects on the gut microbiota, gut-microbiota-derived fecal metabolites, intestinal barrier function and lipid metabolism.^18^ The IHTG content was decreased in exercised rats, in line with lower histology scores for steatosis. Also, exercise downregulated significantly hepatic mRNA expression of liver X-receptor alpha, *Srebp1c, Cepba* and *Cd36* indicating improvements in hepatic lipid metabolism. Strikingly, no significant effect of exercise on the NAFLD activity score (NAS) was detected. Yet, significant improvements in liver enzymes were detected in high-fat-diet exercised rats compared with non-exercised rats, which indicate improvements in inflammation and liver damage. The total gut microbial community and metabolome were changed by the exercise protocol. Interestingly, the high-fat diet increased the alpha diversity (Shannon diversity index) which was reduced to the pre-high-fat diet level after the exercise intervention. Also, it was demonstrated that a high-fat diet in rats increased the *Firmicutes*/*Bacteroidetes* ratio, which was corrected by physical activity. Especially, the relative abundances of *Proteobacteria* and *Bacteroidetes* phyla were increased and the relative abundances of *Verrucomicrobia* phylum was decreased after exercise when compared between high-fat-fed rats with or without exercise. Relative abundances of the *Alkaliphilus, Prevotella* and *Desulfovibrio* genera were increased in exercised high-fat-fed mice compared with non-exercise counterparts.

Moreover, the above discussed study also investigated the gut permeability and its function, plasma LPS, and induced pathways in the liver.^18^ Exercised rats fed with high-fat diet showed downregulation of hepatic and intestinal toll-like receptor 4 (TLR4), tumor necrosis factor 6 (TNF-6) and interleukin 6 (IL-6) mRNA expressions, and importantly, decreased plasma levels of LPS, and improved intestinal barrier function compared with non-exercised rats. Exercise resulted in significantly increased intestinal mucus layer and the upregulation of genes coding intestinal tight-junction proteins, including occluding and claudin-1. These results may indicate that exercise restored leaky gut, resulting in decreased endotoxemia, and LPS caused inflammation in the liver and gut. Also, beneficial effects were seen on bile acid metabolisms in altered fecal bile acids and in the expression of intestinal FXR when high-fat-fed rats were introduced to exercise. In respect to fecal metabolome, other fecal metabolites such as SCFAs, free fatty acids, and different amino acids were also detected and correlated with the gut microbial composition differentially depending on the group. These results suggest that exercise does not only affect the liver lipid metabolism, but also directly, the gut permeability, gut microbiome, and derived metabolites. In conclusion, these results support the beneficial role for exercise reducing NAFLD *via* an effect on the functions of the gut microbiome.

In a mouse study, exercise has been found to increase the abundance of bacteria, in particular *Akkermansia* and *Enterobacteria*, upon exercise.^121^ These bacteria are considered as butyrate-producing bacteria, reflected in increased butyrate levels in the gut which, therefore, could potentially reach the liver and contribute to hepatic lipid metabolism. The study also aimed to show that the exercise and butyrate administration mediated *via* gut microbiome may reduce the liver weight and affect hepatic genes that encode proteins involved in lipogenesis. However, the study did not study the effects on liver lipids, only absolute liver weight.

**Figure 1.**
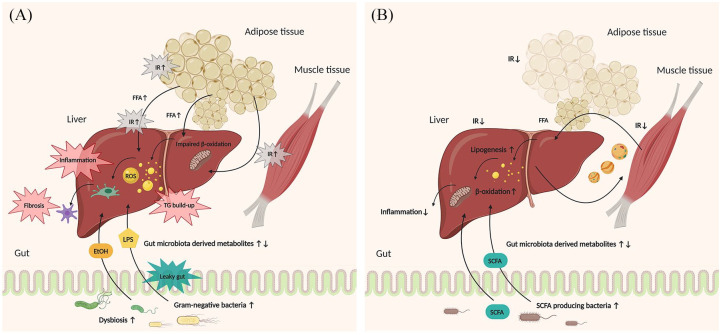
Mechanisms by which exercise and gut microbiome may affect NAFLD. (A) Sedentary situation. Overview of the pathogenesis of NAFLD and the relationship of the gut microbiome in the pathogenesis. A sedentary lifestyle has led to the accumulation of TGs in the liver, due to IR in the peripheral organs and the liver itself, resulting in the production of ROS, and therefore, inflammation and further fibrosis. Dysbiosis and increased gut permeability allow LPS derived from gram-negative bacteria and ethanol synthesized from EtOH-producing bacteria to reach the liver through the portal vein and drive lipid accumulation and inflammation in the liver. (B) Physically active situation. Simplified overview of the beneficial effects of exercise and the gut microbiome on NAFLD. IR is ameliorated by exercise increasing insulin sensitivity, and normalizing lipid metabolism in peripheral tissues and in the liver. Exercise affects the gut microbiota composition, and this increases the abundance of SCFA-producing bacteria and thereby increases the production of SCFAs that can reach the liver. EtOH, ethanol; FFA, free fatty acids; IR, insulin resistance; LPS, lipopolysaccharide; NAFLD, non-alcoholic fatty liver disease; NASH, non-alcoholic steatohepatitis; ROS, reactive oxygen species; SCFA, short-chain fatty acid; TGs, triglycerides.

Altogether, proper knowledge about the mechanisms involved in the effects of exercise on NAFLD in humans is lacking. However, extrapolating the very few rodent studies to the human situation, one can speculate that exercise might have a potential beneficial effect on the gut microbiome and hence gut microbiome might produce metabolites that affect NAFLD and obesity. Figure 1 illustrates the main mechanisms *via* which exercise and the gut microbiome may affect NAFLD. To address the exact mechanisms of exercise and gut microbiome in NAFLD, more studies, both clinical and preclinical, should be employed.

## Discussion

There are multiple mechanisms through which exercise can affect NAFLD, and the gut microbiome is likely a connector in some.

Importantly, several RCTs on exercise on NAFLD find that weight reduction is not the major driver of the reduction of liver fat. The latest literature indicates that exercise is beneficial for lipid metabolism, likely by improving insulin sensitivity, as well as amelioration of inflammation in the liver. Current guidelines of physical activity for patients with NAFLD are based on the evidence that exercise reduces hepatic steatosis and are derived from general recommendations for physical activity. In contrast, current guidelines do not advocate physical activity as a treatment for patients with advanced stages of NAFLD. To our knowledge, only three studies have investigated the effects of exercise on advanced stages of NAFLD, that is, fibrosis, with promising results.^103,110,111^

The majority of studies of the gut microbiome of patients with NAFLD focus mainly on taxonomy level instead of the functional capacities; this is unfortunate because the functional capacities of the microbiome may hold more mechanistic information. Currently, there are not enough available studies with appropriate settings investigating the exercise-mediated effects on the gut microbiome for clear conclusions in patients with NAFLD. The data are mainly based on obese populations, and the results might be unreliable due to questionable study designs. Nevertheless, it can be suggested that exercise has an impact on shaping the gut microbiome, but the effects of this on NAFLD remain unclear.

Since exercise studies show effects on the composition of the gut microbiome, it is expected that these compositional changes may result in differences in metabolites secreted by the gut microbiome, such as the generation of more SCFAs. Of these SCFAs, butyrate has been suggested to affect liver fatty-acid metabolism. Exercise might also affect other gut-derived metabolites, such as ethanol, bile acids, and choline, which have also been shown to be control hepatic lipid metabolism. Another factor that might be affected by exercise and that has not been studied in great detail is endotoxemia.^122,123^

Future clinical studies should be designed as RCTs with sufficient sample size to detect changes in the gut microbiome under different exercise regimens. It is also of importance that research is conducted specifically in patients with diagnosed NAFLD with well described family, medical, and lifestyle history. It would be essential to design the study in a randomized and controlled manner, with aerobic, anaerobic exercise, and non-exercised control groups to see the effect of different types of exercise. A proper control group would need attention; the control group should exclusively include patients with diagnosed NAFLD. Subjects should be randomized by at least age, sex, and ethnicity due to the link between these and NAFLD, but also because of the link to the gut microbiome. The effects of exercise on NAFLD, not only in steatosis but also fibrosis, should be investigated comprehensively with validated instruments and parameters, including non-invasive radiological and magnetic imaging such as MRS and magnetic resonance elastography (MRE), VCTE and/or invasive liver biopsies with histopathological assessment. Also, gut microbiome as an outcome would need proper sampling methods, analytics, and bioinformatics analysis, which are then well described in published methods. If possible, large group sizes with shotgun sequencing is optimal for analyzing the gut microbiome, and especially useful to add mechanistic suggestions to how the variation in microbiome composition may affect the liver.

More mouse studies are needed to elucidate through which pathways the effects of, for example, SCFAs, lipid metabolism, ethanol production and inflammation are driven in NAFLD, with subsequent validation in human studies. It would be interesting to see mouse studies with transplantation of the bacterial species that are suggested to affect NAFLD, to introduce some more causal evidence of the effect of the microbiome. In addition to this, further studies into ethanol levels in human populations with NAFLD, compared with subjects with obesity or diabetes, are appropriate to elucidate whether endogenous ethanol is a major player in the pathophysiology. There are currently a lot of studies on the effect of SCFAs, but we believe the field needs to demonstrate more clearly the effect sizes, and especially the long-term effects, of supplementation of different types of SCFAs. This will further improve the insight into the intriguing interrelated metabolic triangle of physical activity, gut microbiome, and NAFLD.

In conclusion, exercise may affect NAFLD both directly and indirectly *via* effects on the gut microbiome. More causal evidence is needed to further elucidate whether some of the abovementioned mechanisms on the gut–liver axis work in unison, or affect each other. It is paramount that more studies should focus specifically on the mechanisms in NAFLD rather than in obesity. In general, there is a lack of studies which investigate the mechanistic effects of exercise on the gut microbiome, and whether this may influence NAFLD and its progressive stages.
